# Simple, Office-Based Intervention Improves Patient–Provider Relationship in New Patient Hand Visits

**DOI:** 10.1016/j.jhsg.2024.04.002

**Published:** 2024-05-09

**Authors:** Jona Kerluku, Lauren E. Wessel, Jennifer Bido, Claire Isabelle Verret, Duretti Fufa

**Affiliations:** ∗ Department of Hand and Upper Extremity Surgery, Hospital for Special Surgery, New York, NY; † Department of Orthopaedic Surgery, University of California, Los Angeles, CA; § Department of Orthopaedic Surgery, Hospital for Special Surgery, New York, NY; ‡ Department of Orthopaedic Surgery, Northwell Health at North Shore University Hospital and Long Island Medical Center, New York, NY

**Keywords:** Communication tools, Hand surgery, Patient engagement, Rapport, Visit agenda

## Abstract

**Purpose:**

Effective patient–doctor communication is linked to improved patient functional and physiological health status, better adherence to physician recommendations, and increased patient satisfaction. However, studies show that patients have difficulty understanding and recalling information discussed during a medical encounter. The purpose of this study was to assess patient engagement, patient–doctor communication, and patient–doctor interactions with the utilization of a patient encounter card to help aid in communication.

**Methods:**

New patients presenting to a single hand surgeon during an 8-month period between 2019 and 2020 were recruited for this study. Patients were recruited in pre and postintervention phases, defined by the rollout of a patient encounter card. Patients studied in the preintervention group were defined as the control population and experienced a typical office visit. The postintervention group experienced a typical office visit with the addition of a patient encounter card distributed to patients prior to meeting with the physician and screened by the physician during the visit to guide the encounter. Patient satisfaction and engagement surveys were collected during patient checkout process.

**Results:**

Two hundred eighty-seven patients (70% participation rate) were enrolled in the preintervention (145) and postintervention (142) phases. The utilization of a patient encounter card for setting a visit agenda resulted in a significant increase in self-reported patient engagement, improving from 74% to 88%. In both phases, 98% of patients felt that the physician listened well or very well and reported high levels of confidence in the provider being able to address their primary health concerns (72% and 79%, respectively). Overall, patient satisfaction was maintained pre and postintervention (96% and 98%, respectively).

**Conclusions:**

Use of the encounter card improved patients’ feelings of engagement during their visits. Further research is required to determine the impact of these tools on providers’ engagement and patient outcomes to improve quality of care in hand surgery.

**Type of study/level of evidence:**

Therapeutic II.

A foundational element in providing quality clinical care in hand surgery is effective communication between physicians and patients.^1^^,^^2^ Literature has demonstrated that good communication improves both physician and patient satisfaction in clinic, influences the degree of details patients share about their condition, reduces medical costs, and increases likelihood of adherence to treatment.^1^, ^2^, ^3^, ^4^, ^5^, ^6^, ^7^, ^8^, ^9^ Moreover, success in quality communication with a hand surgeon leads to patients feeling greater agency toward their overall health, simultaneously building the rapport needed for future health interactions.^2^^,^^8^

In an increasingly pressured health care landscape, insufficient time with patients in clinic has been identified as one of the major barriers to effective communication.^4^ Family medicine physicians spend an average of 9 to 24 minutes with patients; however, almost one-third of patients do not feel that this is enough time to discuss their concerns.^9^ Across multiple specialties, physicians are responsible for increasingly burdensome administrative roles related to insurance claims, in addition to patient care, causing physicians to spend less time with patients.^7^

The consequences of physicians spending less time with patients have also been evaluated in surgical specialties.^9^ In orthopedic surgery specifically, patient outcomes and satisfaction have been demonstrated to be more dependent on patient comprehension of interactions.^8^, ^9^, ^10^ In a study asking patients to recall when return to normal activity was recommended, the authors found that only 58% understood what was discussed during the medical encounter; meanwhile, 95% of physicians believed patients understood their explanations.^11^ Studies have also shown that 40% to 80% of the medical knowledge provided by health care professionals is forgotten immediately after a medical visit and almost half of what is remembered by patients is incorrect.^1^ Patients with lower health literacy are additionally less likely to ask questions during the medical encounter, contributing to misunderstandings and shorter medical visits.^6^^,^^12^ In hand surgery, specifically, quality of time spent with the surgeon was found to be a better predictor of patient satisfaction than the number of encounters or duration of visit.^8^

Rapport building, upfront collaborative agenda setting, and co-creating a plan of care are powerful strategies to empower patients in hand surgery clinics to make more informed decisions about their care and participate in a medical conversation based on shared understanding.^1^^,^^11^^,^^13^, ^14^, ^15^, ^16^, ^17^ We developed a Patient Encounter Card (PEC) to facilitate patient–doctor collaboration by allowing patients to set the agenda for their hand surgery clinic visits. This PEC allowed patients to identify their greatest concerns at the start of the clinic visit and also served as a semistructured communication tool for orthopedic trainees to use without delaying care during a high-volume hand clinic.

The purpose of this study was to evaluate the impact of the PEC on patient satisfaction and feelings of engagement in a hand surgery clinic. We hypothesized that patients who use the PEC will report an increased connection with their physician and higher satisfaction with their clinic visits.

## Methods

### Study design and patient selection

This was a quality improvement study in a single hand surgery clinic. The study design was approved by the institutional review board. All new patients attending the clinic of a single fellowship-trained orthopedic hand and upper extremity surgeon were recruited. This was a tertiary academic hospital-based clinic with rotating residents and hand fellows, staffed by one attending and nurse practitioner. All new patients with an appointment at the hand clinic were eligible to participate. Data were collected for a total of 8 months in the period between July 2019 and October 2020 (paused for COVID-19 pandemic). Patients presenting for follow-up appointments and age less than 18 were excluded.

### Intervention and data collection

**Figure 1:**
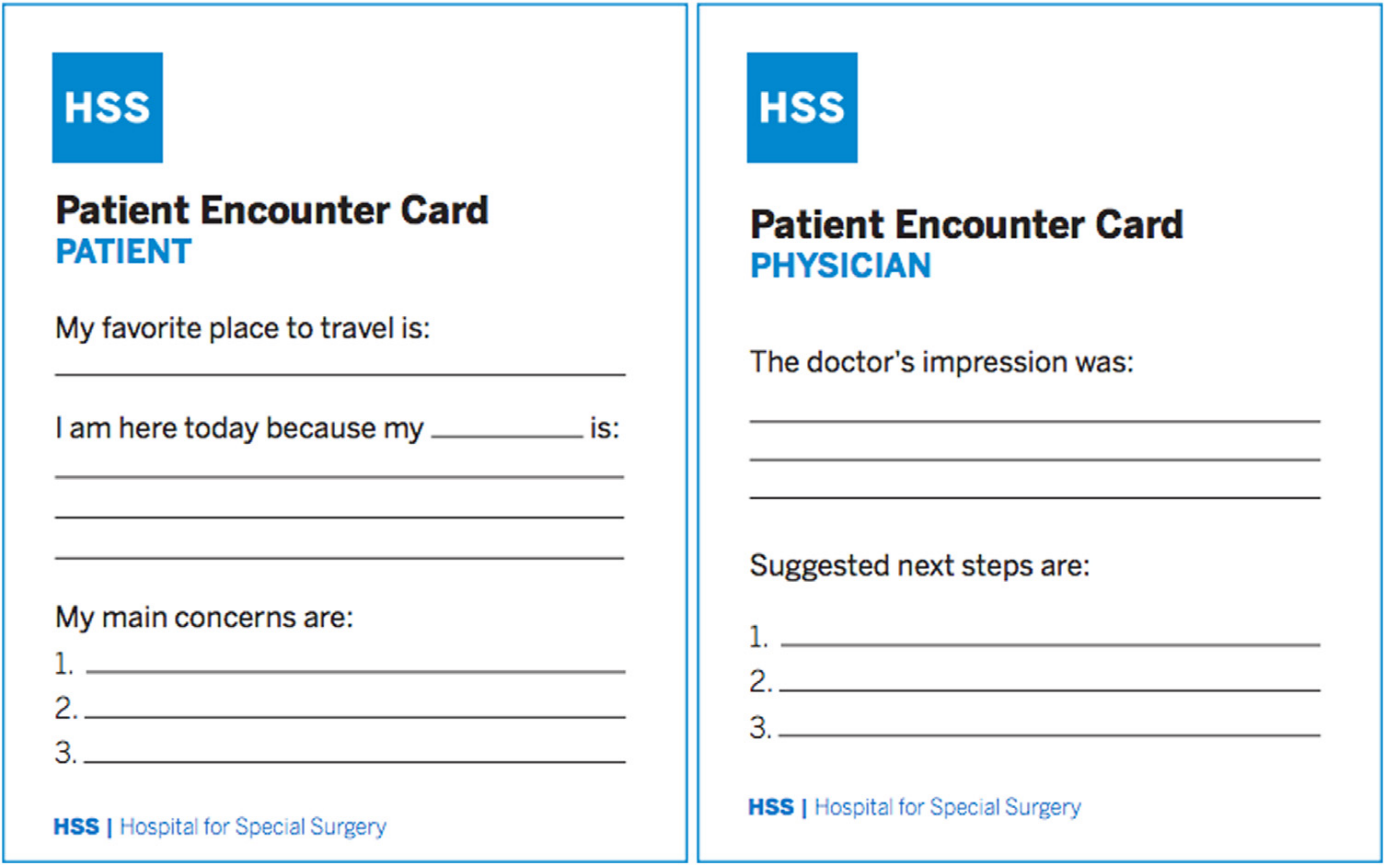
Patient encounter card.

Patients were split into two groups: pre and postintervention. The control group consisted of new patients presenting prior to implementing use of the encounter card. The intervention group consisted of new patients presenting after implementation of the encounter card (Fig. 1). All patients completed the same patient satisfaction and engagement surveys at the completion of their visit during checkout (Appendix S1, available online on the *Journal’s* website at https://www.jhsgo.org).

Eligible patients were called the day prior to their hand clinic appointment to inquire about interest in participating in this research study. Patients who verbally consented were given this card upon arrival for their clinic visit and expected to complete the “Patient” side prior to entering their assigned clinic room. The PEC asked for the following: (1) a personal detail (ie, their favorite place to travel), (2) the purpose of their visit, and (3) a bullet point list of the concerns they would like addressed (Fig. 1). The card was designed to help orthopedic surgery trainees (residents and fellows) and patients in (1) building rapport, (2) setting a collaborative visit agenda upfront, and (3) co-creating a plan of care. The card was then presented to the attending physician, trainees (a resident or fellow), or the nurse practitioner who were expected to address each item on the “Patient Encounter Card” during the visit. At the end of their visit, providers completed the back of the card (Fig. 1), which summarized their visit by stating the physician’s impression and recommendations.

Baseline patient demographics were collected using an anonymous questionnaire. All patients pre and postintervention were also asked to complete a 5-point Likert-scale survey to assess patient engagement and patient–doctor communication (Appendix S2, available online on the *Journal’s* website at https://www.jhsgo.org). The 5-item Perceived Efficacy in Patient–Physician Interaction questionnaire was utilized to assess patient–doctor interaction.^5^ Additionally, feedback was also collected from all trainees at the end of each clinic day.

### Statistical analysis

Descriptive statistics, such as means with ranges and frequency statistics, were used to report baseline characteristics. Differences in categorical variables were detected using chi-square or Fischer exact tests as indicated. Statistical significance was set at α ≤ 0.05.

## Results

### Patient demographics

Two hundred eighty-seven of 400 (70%) eligible patients were successfully recruited for this study and completed all surveys. Data for 145 patients in the control group and 142 in the intervention group were collected, with 140 patients analyzed in each group due to incomplete data for seven patients. Overall, there were more women (56%), the mean age was 49.6 years, 71% of patients were identified as Caucasian, and 43% had graduate degrees. The majority of patients (58%) described their state of health as good. There were no differences in patient demographics between the intervention and control groups (Table 1).

**Table 1:** Demographics of Patients Using Patient Encounter Card at First Clinic Visit

	Baseline (*n* = 145)	Intervention (*n* = 142)	*P* Value
Sex % (*n*)			
Female	56.6 (82)	55.6 (79)	.876
Mean age			
Years	48.3	50.9	.227
Race/ethnicity % (*n*)			
Asian	7.8 (11)	5.7 (8)	
Black or African American	7.1 (10)	6.4 (9)	
Hispanic or Latino	9.2 (13)	9.9 (14)	
White	72.3 (102)	73.0 (103)	
Other	3.5 (5)	5.0 (7)	.793
Education %(*n*)			
No/some college	21.5 (31)	17.2 (24)	
College graduate	36.1 (52)	38.6 (54)	
Graduate school	42.4 (61)	44.3 (62)	.412
Self-reported Health status %(*n*)			
Fair or less	7.6 (11)	6.4 (9)	
Good	60.0 (87)	56.4 (79)	
Excellent	32.4 (47)	37.1 (52)	.636

### Patient experience

Overall, patients in both groups felt satisfied (>95%) with their care and 98% of patients felt that physicians listened well or very well during the conversation (Table 2). Both groups felt that their concerns were addressed (>96%) and that their physician’s recommendations were understandable (>96%).

**Table 2:** Patient Questionnaire Responses

Questions	Baseline % (*n*)	Intervention % (*n*)	*P* Value
The doctor carefully listened to what I had to say.	Well or Very Well 97.9 (141)	Well or Very Well 97.9 (137)	.958
The doctor addressed my main concerns during this visit.	Well or Very Well 96.5 (138)	Well or Very Well 98.5 (138)	.534
The doctor explained the recommendations for treatment in a way I could understand.	Well or Very Well 96.6 (139)	Well to Very Well 99.3 (137)	.279
I was involved in decisions about my treatment plan as much as I wanted to be.	Strongly Agree 74.1 (106)	Strongly Agree 87.8 (122)	**.015**
Know what questions to ask your doctor.	Very Confident 58.3 (77)	Very Confident 62.9 (88)	.895
Make the most of your visit with your doctor.	Very Confident 62.9 (83)	Very Confident 64.3 (90)	.321
Get your doctor to answer all of your questions.	Very Confident 68.2(90)	Very Confident 68.3 (95)	.338
Get your doctor to take your primary health concern seriously.	Very Confident 73.5 (97)	Very Confident 77 (107)	.737
Get your doctor to do something about your primary health concern.	Very Confident 71.8(94)	Very Confident 79.1 (110)	.449
I connected with the doctors.	Well to Very Well 95.9 (138)	Well to Very Well 97.8 (137)	.690
Satisfaction with visit.	Satisfied to Very Satisfied 95.9 (139)	Satisfied to Very Satisfied 97.8 (137)	.501

Bold values indicate statistical significance (*P* ≤ .05).

In terms of their own contribution to the hand clinic visit, fewer patients felt very confident pre and postintervention about their ability to get their doctor to take their concerns seriously (>73%) and answer their questions (68%). Notably, there was a significant increase in patient engagement in the intervention group, improving from 74% to 88% (*P* = .02), as more patients strongly agreed to be involved in decisions about their treatment (Table 2).

## Discussion

This study demonstrated an improvement in patient engagement and involvement with decisions of clinical care after intervention of a PEC for new patient encounters presenting to a hand surgeon. In the era of electronic medical records and brief clinic visits, this study presents one simple intervention that physicians can use to build relationships efficiently and effectively with their new patients.^11^, ^12^, ^13^, ^14^, ^15^, ^16^, ^17^, ^18^, ^19^ Directed conversations and focused content are essential to a physician’s ability to make thorough diagnoses and develop effective treatment plans.^20^

Although many orthopedic providers believe their patients to be well-informed after visits, literature has demonstrated otherwise. A 2015 arthroscopy study by Brophy et al^21^ reported that the average orthopedic patient answered between 49% and 50% of questions correctly, regardless of how they self-rated their knowledge base. Similarly, a study of carpal tunnel patients demonstrated only 75% understanding of general principles of carpal tunnel syndrome and its related surgical treatment after standardized education.^14^ With the understanding and acknowledgment that even with focused patient education, patients continue to lack comprehension of pertinent components of their care, it is even more important that orthopedic surgeons engage patients during their clinic visits. The utilization of our PEC in a high-volume teaching clinic of a single hand surgeon significantly improved patient engagement throughout the course of our study and was evaluated to be a practical added tool across subspecialties based on trainee feedback.

Overall, health care systems are continuing to develop strategies that enhance communication between patients and health care providers.^4^^,^^10^ However, these efforts can be difficult to implement in an era of shorter clinic visits and increasing digitization of health care.^22^ Our study offers a framework that could improve the quality of clinical visits and potentially offer a model for facilitating communication in hand and orthopedic clinics. The essential elements for effective conversations to support patient-centered care have been conceptualized through the 6-Function Model.^22^ This model was replicated in the design of our PEC tool as it provides a framework for medical encounters that encourage rapport building, gathering and providing information, clinical decision making, and responding to patient emotions. Our PEC tool also supported patient communication by allowing patients an opportunity to emphasize their clinical priorities resulting in a significant improvement in their sense of agency toward developing a treatment plan with their physician. Prior research has demonstrated that successful communication and cocreation of a treatment plan positively impacts patient compliance and overall quality of care as patients engaged in their care plan have better adherence, outcomes, and overall satisfaction.^1^, ^2^, ^3^, ^4^, ^5^^,^^22^ It is important to note that our study was conducted within a tertiary academic hospital-based hand clinic where trainees are a vital component in providing quality orthopedic care. A recent 2023 *JBJS* study by Shing et al revealed that only about 22% of orthopedic surgery programs provide formal health literacy training but of residents receiving training in communication, up to 98% found it to be effective.^23^ The PEC tool reported in our study could also serve as a resourceful educational instrument to support interpersonal and communication skill competencies within training programs.

Further research is necessary to determine whether the improved patient engagement achieved with our PEC contributes to improved long-term outcomes and compliance in patients with hand concerns. Although not statistically significant, patients surveyed postintervention also demonstrated a trend toward greater satisfaction and connection with their doctors. Given that our patients demonstrated generally high satisfaction even preintervention, reproducing the study in a setting where baseline patient satisfaction is known to be lower (ie, socioeconomically underserved populations) could reveal a more significant impact and may be an important subject of future study.^24^, ^25^, ^26^ This synergistic relationship of physician perceived quality of care through improved communication and patient satisfaction has been further shown to have a significantly positive impact on all three dimensions of burnout (emotional exhaustion, depersonalization, and personal accomplishment).^27^

Our study was limited in that it was conducted in a patient population with high preintervention metrics, such as satisfaction. For example, our patient questionnaire revealed statistically significant findings in only one area: “I was involved in decisions about my treatment plan as much as I wanted to be.” Our metrics might have demonstrated more significant differences pre and postintervention had the PEC been applied in practices with less uniform preintervention patient satisfaction such as that in spine or trauma care.^28^^,^^29^ Additionally, given the practice demographics, it can be assumed that the health literacy among the assessed patients is above average, indicating potential sample size limitations. We note that effects of our intervention may be different in varying patient populations, which would be an important area of future study. Finally, we did not measure the time required for the patients to fill out this card but do acknowledge that this may add to the administrative burden for both patients and office staff. Future research may consider digital adaptations of this tool in supporting provider clinical efficiency and generalizability of our tool toward improving the quality of patient–physician communications within health care systems, especially in hand surgery.

In conclusion, we utilized a PEC designed to address the main concerns of patients with their treating providers. Patients rated encounters as being more collaborative postintervention, affording a sense of agency in their hand clinic visits. Our results are promising in showing a significant increase in patient engagement and maintaining high patient–doctor communication, interaction, connection, and overall patient satisfaction. All of which contribute to building a healthy patient–doctor relationship.
