# Supplementary material for: Simple, Office-Based Intervention Improves Patient–Provider Relationship in New Patient Hand Visits
Source: J Hand Surg Glob Online. 2024 May 9;6(4):529–33. doi: 10.1016/j.jhsg.2024.04.002 (PMC11331220; doi:10.1016/j.jhsg.2024.04.002)
Supplement: Appendix S1 [file mmc1.docx]

**Appendix 1. Adapted^5^ Pre-and Post-Intervention Questionnaires**

1. Age: ____
2. Sex

- Female
- Male

1. Race/Ethnicity

- Asian
- Black or African American
- Hawaiian or Pacific Islander
- Hispanic or Latino
- White
- Other

1. Education level

- No college
- Some college
- College graduate
- Graduate school

1. Employment status

- Employed/Self-Employed
- Homemaker
- Retired
- Unable to work
- Unemployed

1. How would you describe your general health?

- Very Poor
- Poor
- Fair
- Good
- Excellent

1. Is this your first visit in Dr. [Senior Author]’s office?

- Yes
- No

1. Did you use the “My Ortho Visit” patient encounter card with your doctor today?

- Yes
- No

1. Please tell us who you spoke to during your visit today. Check all that apply.

- [Senior Author]
- Resident Physician/ Fellow
- Nurse Practitioner

1. Rate your experience in clinic today:

- Very Satisfied
- Satisfied
- Neutral
- Dissatisfied
- Very Dissatisfied

1. The doctors carefully listened to what I had to say:

- Very well
- Well
- Fairly
- Poorly
- Very poorly

1. The doctors addressed my main concerns/questions during this visit (i.e. the doctors focused on what was important to me):

- Very well
- Well
- Fairly
- Poorly
- Very poorly

1. The doctors explained the reasons/recommendations for treatment in a way I could understand:

- Very well
- Well
- Fairly
- Poorly
- Very poorly

1. I was involved in my treatment plan as much as I wanted to:

- Strongly agree
- Agree
- Neutral
- Disagree
- Strongly disagree

1. I needed more time to discuss my health concerns/questions:

- Strongly agree
- Agree
- Neutral
- Disagree
- Strongly disagree

1. I trusted/had confidence in the doctors:

- Strongly agree
- Agree
- Neutral
- Disagree
- Strongly disagree

1. I connected with the doctors:

- Very well
- Well
- Fairly
- Poorly
- Very poorly
