# Supplementary material for: Simple, Office-Based Intervention Improves Patient–Provider Relationship in New Patient Hand Visits
Source: J Hand Surg Glob Online. 2024 May 9;6(4):529–33. doi: 10.1016/j.jhsg.2024.04.002 (PMC11331220; doi:10.1016/j.jhsg.2024.04.002)
Supplement: Appendix S2 [file mmc2.docx]

**Appendix 2. Adapted^3^ Perceived Efficacy in Patient-Physician Interaction**

For the following questions, please indicate how confident you are on a scale of 1 to 5.

With 1 being “Not at all confident” and 5 being “Very Confident”.

**How confident are you in your ability to:**

1. Know what questions to ask your doctor
2. Get your doctor to answer all of your questions
3. Make the most of your visit with your doctor
4. Get your doctor to take your chief health concern seriously
5. Get your doctor to do something about your primary concern
